# Secondary Ion Mass Spectral Imaging of Metals and Alloys

**DOI:** 10.3390/ma17020528

**Published:** 2024-01-22

**Authors:** Yanjie Shen, Logan Howard, Xiao-Ying Yu

**Affiliations:** 1 College of Biology and Oceanography, Weifang University, 5147 Dongfeng East Street, Weifang 261061, China; 2 Materials Science and Technology Division, Oak Ridge National Laboratory, Oak Ridge, TN 37830, USA; 3 The Bredesen Center, 310 Ferris Hall, 1508 Middle Drive, Knoxville, TN 37996, USA

**Keywords:** Secondary Ion Mass Spectrometry (SIMS), static SIMS, dynamic SIMS, imaging, mentals, alloys

## Abstract

Secondary Ion Mass Spectrometry (SIMS) is an outstanding technique for Mass Spectral Imaging (MSI) due to its notable advantages, including high sensitivity, selectivity, and high dynamic range. As a result, SIMS has been employed across many domains of science. In this review, we provide an in-depth overview of the fundamental principles underlying SIMS, followed by an account of the recent development of SIMS instruments. The review encompasses various applications of specific SIMS instruments, notably static SIMS with time-of-flight SIMS (ToF-SIMS) as a widely used platform and dynamic SIMS with Nano SIMS and large geometry SIMS as successful instruments. We particularly focus on SIMS utility in microanalysis and imaging of metals and alloys as materials of interest. Additionally, we discuss the challenges in big SIMS data analysis and give examples of machine leaning (ML) and Artificial Intelligence (AI) for effective MSI data analysis. Finally, we recommend the outlook of SIMS development. It is anticipated that in situ and operando SIMS has the potential to significantly enhance the investigation of metals and alloys by enabling real-time examinations of material surfaces and interfaces during dynamic transformations.

## 1. Introduction

Secondary Ion Mass Spectrometry (SIMS) is a powerful mass spectral imaging (MSI) technique, and it has been extensively employed for comprehensive surface analysis and characterization of materials [1,2,3,4]. Its root traces back to 1910 [5], and its early applications are in inorganic materials and semiconductors. During SIMS analysis, a high-energy primary ion beam bombards the solid surface. This interaction with the surface induces the emission of secondary ions (SIs), different from the primary ions, as well as neutral particles. These emanations originate from the top few layers of atoms at the surface. Subsequently, a mass spectrometer analyzes the extracted secondary ions, providing valuable insights into the composition and structural characteristics of the material composition. In contemporary applications, SIMS has evolved into an indispensable tool across diverse fields, such as materials research, medical research, geology, cosmochemistry, and the life sciences [6,7]. Although the original invention of SIMS lies heavily in solid, inorganic, and metal materials, its capability to unravel the intricate details of elemental, molecular, and isotopic components makes SIMS valuable in advancing scientific inquiry and technological development of materials.

SIMS stands out prominently for using ion beams among MSI techniques. Renowned for its high mass resolution, high spatial resolution imaging, and depth profiling capabilities, SIMS is widely used in scrutinizing the local distribution of elements within metals and alloys. While several other techniques, namely transmission electron microscopy coupled with energy-dispersive X-ray spectroscopy (TEM/EDX), analytical scanning electron microscopy coupled with energy-dispersive X-ray spectroscopy (SEM-EDX), and atom probe tomography (APT), have all been crucial in metal and alloy research, SIMS distinguishes itself from others and offers unique advantages [8]. Both TEM/EDX and SEM-EDX permit imaging of sample nanostructures coupled with elemental analysis. APT boasts remarkable spatial resolution in three dimensions (3D); however, its extensive sample preparation requirements and limited material volume pose challenges to revealing the complete structural information of materials. Specifically, APT analysis achieves sensitivities in the ppm level for specific analytes. Notably, SIMS provides higher detection sensitivity than APT, generally exhibiting detection sensitivities at the ppm to ppt level. SIMS also gives reasonable spatial resolution (at the sub-micrometer level) and offers the ability to analyze small surface areas. It excels in detecting a wide array of elements, encompassing both metals and non-metals, thereby presenting compelling solutions compared to other bulk and microanalysis approaches. Moreover, SIMS has impressive capabilities, including the ability to generate depth profiles with excellent detection sensitivity, depth resolution, composition, and impurity measurements (e.g., metals, dielectrics, semiconductors). Additionally, it offers an outstanding dynamic range, reaching up to six orders of magnitude. This multifaceted capability makes SIMS a versatile and powerful tool in materials research and microanalysis.

The SIMS technique unfolds along two distinct paths, namely static SIMS and dynamic SIMS. These divergent approaches exhibit disparities in primary ion beam operation modes and mass analyzer configurations [9]. In the realm of static SIMS, a prevalent choice is the Time-of-Flight SIMS (ToF-SIMS) instrument, and the pulsed primary ion beam is within the energy range of a few keV to tens of keV. Conversely, dynamic SIMS operates with a substantially higher primary ion current, with NanoSIMS as an outstanding example.

**Figure 1:**
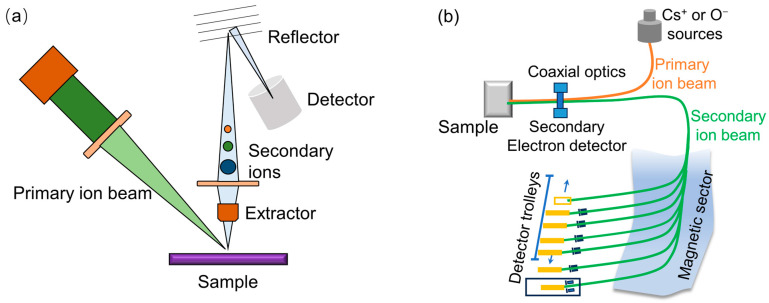
The schematics of ToF-SIMS (**a**) and magnetic SIMS (**b**) to depict the main differences between the two main types of SIMS instruments.

Noteworthy distinctions emerge in the types of primary ions utilized, while ToF-SIMS often employs either single-atom (i.e., Bi^+^, Au^+^) or clustered primary ions (i.e., Bi_n_^+^, Au_n_^+^, Ar_n_^+^, C_60_^+^). NanoSIMS, a type of magnetic SIMS, typically relies solely on single-atom primary ions. In the analytical domain, ToF-SIMS excels by furnishing information encompassing the full spectral information, including isotopic, elemental, molecular ions, fragments of larger molecules, or clusters of ions. In contrast, magnetic SIMS often has a higher sensitivity to selected ions. For example, NanoSIMS specializes in elemental and isotopic composition analysis with 5 or 7 detectors. The schematics of ToF-SIMS and magnetic SIMS are shown in Figure 1 to depict the main differences between the two main types of SIMS instruments.

There has been a rapid and noteworthy evolution in SIMS instrumentation in the past decade. For example, Physical Electronics (PHI) and IONTOF GmbH (IONTOF), two front runners of ToF-SIMS manufacturers, made great efforts to the development of ToF-SIMS tandem mass spectrometry instruments (MS/MS) [10,11,12,13,14,15,16,17,18]. This strategic innovation addresses the limitations of traditional ToF-SIMS, notably enhancing the identification of high-mass fragments (>200 Daltons) that have the same mass-to-charge ratios (*m*/*z*) but different structures. Different from the ToF-ToF tandem mass spectrometry designed in the PHI SIMS, both ToF and Orbitrap^TM^ dual analyzers are employed in the IONTOF MS-MS SIMS. Furthermore, IONTOF spearheaded the integration of Scanning Probe Microscopy (SPM) platforms in the ToF-SIMS instrument [19,20,21,22], representing another milestone in technological synergy and efficient multimodal imaging and analysis within one instrument platform. This coupling has substantially elevated the ability to discern the initial topography of sample surfaces and monitor topographic alterations with heightened accuracy and sensitivity during depth profiling. Such techniques enhance our capacity for detailed and comprehensive material characterization. ZEISS has emerged as a trailblazer through its exceptional strides in the development of Focused Ion Beam SIMS (FIB-SIMS) [23], contributing significantly to the intricate characterization of materials at the nanoscale. This imaging mode SIMS coupled with FIB was based on the development of the Luxembourg Institute of Technology SIMS [24,25,26], which currently lacks high mass accuracy and wide mass range.

This review provides a retrospective account of the historical evolution of SIMS while elucidating recent developments and applications. Additionally, we offer an overview of the fundamental principles underlying SIMS and outline the prevalent instrumentation development and relevant applications in metals and alloys. Subsequently, the review delves into the diverse applications of various SIMS instruments, such as ToF-SIMS, large geometry SIMS, and NanoSIMS, particularly giving examples in microanalysis and imaging of metals and alloys. We then follow with a discussion of the SIMS data challenge and emphasize the use of ML and AI for MSI data analysis. Finally, we give an outlook and recommend that in situ and operando SIMS will offer a great opportunity to improve the analysis of metals and alloys significantly in the future.

## 2. Development of SIMS

### 2.1. History of SIMS

The origins of SIMS can be traced back to the year 1910 when J.J. Thomson revealed the generation of secondary ions in a gas discharge tube. Simultaneously, Thomson discovered isotopes with the same chemical nature but different masses for each element, identifying two isotopes of Ne, namely, ^20^Ne and ^22^Ne. A critical advancement occurred in 1931 when Woodcock obtained negative ion spectra of NaF and CaF_2_ at an approximate integer mass resolution, marking the world’s first known secondary ion mass spectrometry. The inaugural instrument utilizing secondary ions for analytical purposes was described in 1949 by Herzog and Viehböck. They employed a gas discharge tube to generate primary ions and utilized a ‘Thomson parabola apparatus’ for positive secondary ion analysis, focusing primarily on metal targets. Subsequent to this groundbreaking work, during the late 1950s and throughout the 1960s, various analytical SIMS instruments were constructed in both academic and industrial settings [27,28,29,30]. These early SIMS instruments incorporated magnetic fields for the mass separation of generated secondary ions.

In the 1970s, SIMS development diverged into two directions: static and dynamic SIMS. Benninghoven and her collaborators utilized large-spot, low-density ion beams (Static SIMS) to analyze the surfaces of organic samples. In contrast, Wittmaack and Magee et al. employed a high-density primary beam (dynamic SIMS) to obtain longitudinal concentration profiles of inorganic samples and identify trace impurities. The distinction between static and dynamic SIMS lies in the primary ion dose and its impact on sample abrasion. In the case of static SIMS, commonly employed in dedicated ToF-SIMS instruments, the primary ion dose ranges from 10^12^ to 10^13^ ions/cm^2^. This low dose assures that each primary ion strikes an undamaged area, minimizing surface contamination from implanted ions and preventing interference with the SIMS signal.

In contrast, dynamic SIMS employs a significantly higher primary ion current, enabling the capability for depth profiling. This methodology is frequently utilized by magnetic sector SIMS instruments [31]. One such representative is the NanoSIMS, which was developed in the 1990s initially for studying dust in space in astrophysics. It has quickly expanded in biological and medical research domains and has been applied in diverse fields, including material sciences, cosmochemistry, and geosciences. What distinguishes the NanoSIMS from other dynamic SIMS instruments is its unique combination of very high spatial resolution (down to 50 nm) and high collection efficiency, resulting in improved transmission and sensitivity. Furthermore, it boasts sufficient mass resolution to effectively separate most of the crucial isobaric interferences [8]. The initial introduction of quadrupole and ToF analyzers took place in the 1970s and 1980s, respectively. The inaugural International SIMS Conference took place in Germany in 1977, becoming a biennial event over three decades to reflect the ongoing progress in SIMS development and applications [5].

Since the inception of the first commercial SIMS in 1961, SIMS has found widespread application across diverse fields. Over the past two to three decades, SIMS methodology has experienced rapid development, achieving detection sensitivity in the range of ppm to ppt. The scope of analysis spans metals, semiconductors, multilayers, organics, thin films, single cells, and biofilms, contributing to fundamental research in chemistry, physics, biology, and microbiology. Additionally, SIMS has expanded into practical domains such as microelectronics, metallurgy, ceramics, earth and space sciences, and applications in medicine and bioengineering.

### 2.2. Recent Development of SIMS

In traditional ToF-SIMS analysis, identifying the composition of high-mass fragments (>200 Daltons) can sometimes pose challenges in terms of unambiguous identification. To address this limitation, PHI has actively developed a ToF-SIMS tandem MS/MS. This innovative instrument enables the selection of secondary ions of interest (precursor ions) for further detailed study and identification of masses [10]. In the MS/MS mode, the chosen precursor ion is directed into a high-energy Collision Induced Dissociation (CID) cell. Within the CID cell, the precursor ions undergo collisions with argon gas atoms, leading to the fragmentation of precursor ions. The resulting fragmented ions are subsequently mass-separated in a linear ToF analyzer and recorded by a second pulse counting detector (MS2), while the remaining ions are collected as usual with the MS1 detector. Once again, a complete mass spectrum is rapidly gathered for each image pixel using the MS2 detector. Operating the ToF-SIMS in the MS/MS mode not only enhances sensitivity for species with mass interferences but also proves beneficial in cases where the peak of interest is affected by other compounds. By detecting a distinctive MS/MS fragment ion associated with the species of interest, the limit of detection can be significantly improved. IONTOF also provides an MS/MS solution for the M6 and M6 Hybrid SIMS systems. IONTOF provides different tools such as spectra libraries, a fully integrated Multivariate Statistical Analysis (MVSA) software package, and the ultimate performance Orbitrap^TM^ extension. This kind of extension is different from the ToF-ToF tandem mass spectrometry designed in PHI [32]. With the TOF MS/MS option, IONTOF also offers a cost-effective MS/MS solution for the M6 model. This option is ideally suited for quick confirmation of anticipated contaminants or compositions and fast MS/MS imaging or depth profiling applications.

In 3D SIMS depth profiling, discerning the initial topography of the sample surface and monitoring topographic alterations during measurement pose a challenge [25]. Complementary insights into surface topography and the ability to gauge the physical characteristics of the analyzed sample can be obtained using in situ SPM within the same ToF-SIMS platform. By seamlessly integrating SPM and SIMS, genuine in situ 3D chemical imaging becomes achievable, seamlessly merging the top-tier performance of the SIMS with the capability to conduct in situ SPM measurements [19,20]. The expansive SPM unit covers a scan range of up to 80 × 80 × 10 µm^3^, making it exceptionally well-suited for furnishing topographic details essential for authentic 3D SIMS measurements.

The inception of FIB-SIMS arose from the initial limitations faced by traditional SIMS in sample preparation and imaging resolution [31]. In FIB-SIMS, a focused ion beam is meticulously directed onto the sample surface, facilitating precise control over material removal and milling. This capability empowers the generation of meticulously defined cross-sections, trenches, or 3D structures with nanoscale precision. The collaborative synergy between FIB and SIMS significantly amplifies the spatial resolution and depth profiling capacities inherent in SIMS analysis. Currently, ZEISS is the only vendor that offers a commercial FIB-SIMS [31], particularly in the realm of high-sensitivity nano-scale materials analysis. The recent technological development of SIMS capabilities positions the scientific community at the forefront of cutting-edge analytical techniques for intricate material characterization.

## 3. SIMS Principles

SIMS is an exceptionally sensitive surface chemical analysis technique with remarkable capability to discern elemental, isotopic, and molecular composition from the very first few atomic layers, typically at or near the sample surface. The core of the SIMS technique lies in the generation of SIs, an essential process that underpins its analytical power. First, a primary high-energy beam of ions strikes the surface of samples. The kinetic energy of these primary ions is subsequently imparted to the solid sample, inciting a cascade of collisions within the material. In the wake of these interactions, these collisions culminate in a return to the sample’s surface, leading to the ejection of atoms and clusters from the sample matrix. It is worth noting that most of the particles ejected in this process are electrically neutral, while only a minute fraction (approximately 1%) of particles carry an electrical charge, which can be either positive or negative. The determination of their charge state is contingent upon the electric field applied between the extractor and the sample. The ensuing step in the SIMS procedure involves the analysis of the *m*/*z* of these ejected species. This analysis gives rise to positive and negative secondary ion mass spectra, respectively, which provide insights into the distribution of ions according to their *m*/*z* values. These spectra graphically depict the ion’s *m*/*z* on one axis and the counts of ions detected at each specific *m*/*z* value on the other. One noteworthy aspect to consider is that the primary ions utilized in SIMS possess considerably high energy. This heightened energy imparts the propensity for extensive fragmentation and reorganization of atoms within the sample. The extent of such fragmentation is directly related to the energy per atom of the primary ion projectile. It is essential to acknowledge that due to the inherent limitations of SIMS, the *m*/*z* values of the ejected secondary ions typically fall below the 1000 mark, as noted in a recent publication [3].

SIMS is a powerful and precise analytical technique that probes the surface composition of materials at a molecular and atomic level, offering invaluable insights into the intricate world of surface chemistry and material analysis. The formation of SIs in SIMS is intricately linked to a range of factors, primarily contingent on the properties of the primary ion beam, including its composition, current density, and energy. The versatility of SIMS stems from the diverse array of primary ion sources at its disposal. The primary ion sources encompass ions, such as Au^+^, Bi^+^, Ga^+^, and Cs^+^, gaseous ions like He^+^, Ne^+^, Ar^+^, Xe^+^, and even atomic or molecular clusters, such as Bi_n_^+^, Au_n_^+^, Ar_n_^+^, or (H_2_O)_n_^+^. Additionally, other ionizing molecules, such as C_60_^+^ and SF_5_^+^, are instrumental in the SIMS process. Among these ion sources, gases such as He^+^, Ne^+^, Ar^+^, and Xe^+^ are particularly noteworthy for their inert nature when interacting with the solid. Their lack of chemical reactivity makes them ideal for applications in SIMS. In contrast, primary ions, like O_2_^+^ and Cs^+^, exhibit chemical reactivity, leading to the creation of electronegative or electropositive surface species on the sample. Consequently, O_2_^+^ ions can significantly enhance the yield of positive ions, while Cs^+^ ions are known to augment the production of certain negative ions. These characteristics render O_2_^+^ and Cs^+^ ions essential in specific SIMS scenarios. In the realm of static SIMS imaging, Ga^+^ ions reign chiefly due to their ability to provide the smallest probe size, typically around 10 nm, with the highest current density (brightness) ranging from 1 to 10 A per cm^2^. However, Ga^+^ ions have limitations, as they are less effective at generating heavy or molecular SIs for *m*/*z* > 500. Consequently, image resolutions smaller than 1 μm are achievable. On the plus side, Ga^+^ ions exhibit inert behavior, preventing the generation of electronegative or electropositive species at the sample surface. Ga^+^ ions also can increase the SIs yield for the same reason. Modern trends in SIMS have seen a shift away from Ga^+^ Liquid Metal Ion Sources (LMIGs) in favor of Bi_n_^+^ and Au_n_^+^ LMIGs. New C_60_^+^ and gold ion sources offer the benefits of high intensity, extended lifetimes, and micrometer-scale spatial resolution. These advancements have revolutionized the field of SIMS analysis [33,34,35]. It is noteworthy that the utilization of polyatomic primary ions, including cluster ions such as SF_5_^+^, Au_n_^+^, Bi_n_^x+^, and C_60_^x+^, has significantly enhanced the yields of molecular SIs when compared to their monoatomic counterparts. Importantly, these primary cluster ion beams are now readily available in commercial SIMS instrumentation, further expanding the capabilities of this powerful technique.

The Giant Gas Cluster Ion Beam-SIMS (GCIB-SIMS), leveraging its capability to capture molecular ions or substantial fragment ions, has demonstrated remarkable potential for mapping intact biomolecules within tissue and cell samples with a much higher mass range [36]. GCIB-SIMS operates by employing giant cluster ions as the primary ion beam, with these clusters typically comprising hundreds or even thousands of atoms and molecules. The utilization of such colossal cluster ions confers several advantages, including heightened sputtering efficiency and enhanced depth resolution in comparison to traditional SIMS methods. Notably, recent applications of various GCIBs, such as CO_2_ and mixed gases like Ar and CO_2_, have proven instrumental in the investigation of organic materials and biological specimens [37,38,39].

### 3.1. Mass Analyzers

The choice of mass analyzer is crucial to the success and precision of the SIMS analysis. There are three prominent types of mass analyzers widely employed in SIMS: ToF, Quadrupole mass analyzer/spectrometer (QMA/QMS), and Double-Focusing Magnetic Sector Analyzers. Each analyzer serves specific purposes, and its selection depends on the requirements of the analytical task. The comparison of the three types of mass analyzers is illustrated in Table 1. QMA/QMS and magnetic mass analyzers are the go-to choices for elemental depth profiling in SIMS due to their unique characteristics. QMAs have garnered popularity for their compatibility with ultrahigh vacuum conditions, making them well-suited for SIMS experiments. These analyzers offer exceptional transmission efficiency, and they are relatively insensitive to the kinetic energy of SIs, rendering them invaluable for both dynamic and static imaging SIMS studies. Moreover, QMAs can be positioned at a considerable distance from the sample target, which facilitates the analysis of larger sample areas. However, QMAs have limitations, notably in their mass resolution, which is typically restricted to around 1 atomic mass unit (u). Additionally, the mass range is constrained to 1–1000 u when using QMAs [40,41].

Magnetic sector instruments, on the other hand, excel in their ability to provide high mass resolving power and exceptional SI transmission, making them highly attractive for precise SIMS analysis of elements and isotopes. Nonetheless, these instruments have their drawbacks. Their ion extraction geometry can hinder the analysis of large sample areas, and there are restrictions for the number of masses that can be simultaneously analyzed. These limitations need to be considered when choosing a magnetic sector mass analyzer for a particular SIMS experiment. Furthermore, it is worth noting that double-focusing magnetic sector mass analyzers are commercially available, particularly within the Cameca series of microprobes [42]. These analyzers come with the added benefit of achieving lateral resolutions of less than 50–150 nm in microanalysis applications and around 1 μm in microscopy setups. This level of spatial resolution is instrumental for high-precision SIMS investigations.

The ToF analyzer is an ideal choice for the static SIMS technique because it combines the high transmission and mass resolution capabilities of the magnetic sector analyzer and the multiplexing abilities of QMAs. Thus, it is possible to perform a time multiplexing of the detection of a few chosen *m*/*z* using QMAs. In contrast, a full spectrum of *m*/*z* peaks is detectable when using the ToF analyzer. One of the key features of ToF analyzers is their exceptional mass resolution, often exceeding 10,000, a metric expressed as *m*/Δ*m* in the spectral mode. This impressive mass resolution allows for discrimination between ions of similar *m*/*z*. Furthermore, ToF analyzers facilitate parallel detection of ions across the entire mass range, which greatly aids the analysis of the higher ions and fragments that have relatively low ion yields typically encountered in static SIMS. 

Two primary advantages underpin the popularity of ToF mass analyzers. First, their multiplexing capability is a remarkable asset. ToF analyzers can sequentially detect all ejected ions, each with distinct mass and flight times. This inherent multiplexing ability significantly boosts the efficiency and throughput of SIMS analyses. Second, the design of ToF analyzers is relatively straightforward, making them both practical and cost-effective. In contemporary SIMS applications, ToF mass analyzers have become widely adopted for static SIMS measurements, particularly in molecular depth profiling. These analyzers excel in their capacity to separate SIs based on their *m*/*z*, while the *m*/*z* of the ions is accurately determined by measuring the time they take to traverse the length (*L*) of the field-free flight tube after being accelerated to a common energy (*E*) in an extraction field. 

In essence, the ToF analyzer leverages the precise measurement of flight times to deduce the mass of ions, enabling high-resolution and high-throughput static SIMS analysis. The flight time depends on the mass weight of ions, which is proportional to the square root of the mass of the secondary ion. As a result, the lighter ions travel faster than the heavier ones and will arrive at the detector earlier.

The main problem of ToF analyzers is measurement uncertainty in ion flight time, and possible sources include SIs creation time and kinetic energy. To address this challenge and to enhance the precision of ToF analyzers, advancements have been made in ion beam technology. These advancements allow for the generation of primary ion pulses with durations of less than 1 ns while maintaining a beam diameter of approximately 100 nm. This reduction in the creation time of primary ions helps mitigate uncertainties associated with the ion’s starting point in the flight path. Furthermore, the utilization of time-to-digital converters (TDCs) represents a significant advancement that greatly improves the accuracy of measuring the flight times of molecular ions. TDCs play a central role in enhancing the overall performance and reliability of ToF analyzers. For ToF analyzers, the best mass resolution is achieved when the energy of the ions remains constant. To compensate for any energy spread within the ion beam, two commonly employed instrument designs come into play, namely high-performance ‘reflection’ configuration and employment of three electric sectors. The latter focuses ions of the same mass but slightly different energy to the detector position.

Recently, several commercial companies and research groups have made a rapid and noteworthy evolution in SIMS technology, such as ToF SIMS V or M6 Hybrid SIMS produced by IONTOF, nanoTOF3 produced by PHI, J105 SIMS produced by IONOPTIKA, NanoSIMS 50L produced by CAMECA, and SHRIMP produced by Australian Scientific Instruments in Canberra, Australia. The comparison of these SIMS products is illustrated in Table 2.

**Table 2:** Differences between commercially mainstream SIMS instruments.

Types of SIMS	Energy of the Primary Ion Source	Primary Ion	Mode of the Primary Ion Source	Mass Analyzer	Spatial Resolution	Sensitive	References
ToF SIMS V (IONTOF)	tens of keV	Ar_n_^+^, Bi_n_^+^, Cs^+^, C_60_^+^, O_2_^+^	Pulse	ToF	<300 nm	ppm to ppb	[43,44]
M6 Hybrid SIMS (IONTOF)	A few keV to tens of keV	O_2_^+^, Ar_n_^+^, Xe^+^, MCs^+^, Bi_n_^+^, Bi_3_^++^	Pulse	ToF and Orbitrap^TM^	<50 nm	ppm to ppb	[45]
PHI nanoTOF3	A few keV to tens of keV	Bi_3_^++^, LMIG with Bi and Au, Ga emitter, Ar_n_^+^, O_2_^+^	Pulse	ToF and ToF	<500 nm in high mass resolution mode <50 nm in high spatial resolution mode	ppm to ppb	[46,47]
J105 SIMS (IONOPTIKA)	A few keV to tens of keV	Ar_n_^+^, (CO_2_)_n_^+^, (H_2_O)_n_^+^ C_60_^+^, C_60_^++^, C_60_^+++^, Au^+^, Au^++^, Au_2_^+^, Au_3_^+^, O_2_^+^, Cs^+^	Continuous	ToF	Tens to hundreds of nanometers	ppm to ppb	[48]
NanoSIMS 50L (CAMECA)	A few keV to tens of keV	Cs^+^, O^−^	Continuous	Magnetic	<50 nm	ppm	[49]
SHRIMP-II	~10 keV	O_2_^−^	Continuous	Magnetic	5–30 μm	ppm	[50,51]

### 3.2. In Situ and Operando SIMS

**Figure 2:**
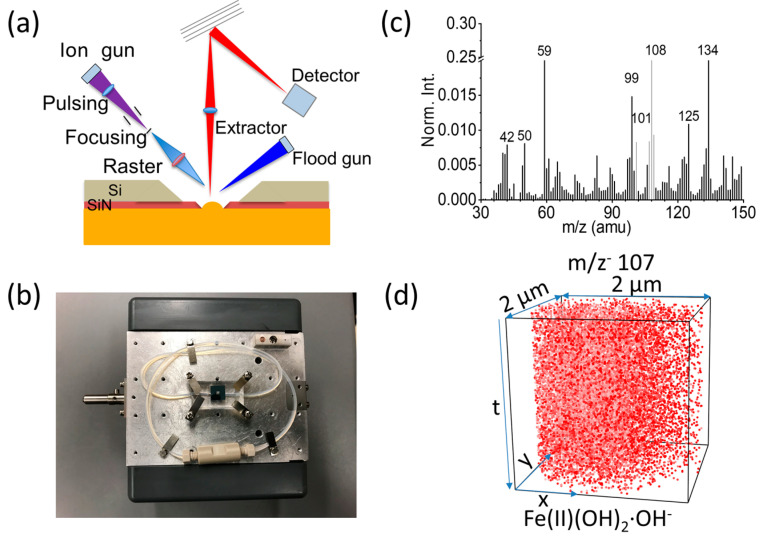
The schematic of GR analysis in liquid: (**a**) the liquid ToF-SIMS schematic setup enabled by SALVI; (**b**) a photo of the SALVI device installed on the sample stage; (**c**) a representative in situ liquid SIMS mass spectrum; and (**d**) a reconstructed 3D image of *m*/*z*^−^ 107 Fe(II)(OH)_2_OH^−^. Reproduced with permission from Ref. [53]. The intensity of red color from light to dark indicates counts. Lighter red color corresponds to lower counts and darker red color higher counts. Copyright 2020 Wiley.

Traditional SIMS often involves static analysis of a sample surface that limits its applicability in understanding materials’ dynamic behavior during various processes. Thus, it is vital to development in situ/operando SIMS to provide real-time investigations of material surfaces as they actively undergo transformations. Regarding the distinction between in situ and operando, it is widely considered that in situ represents a measurement taken in the original position, while operando means a measurement conducted under an ongoing condition [52]. Both are significant methods to enable analysis in real-time, capturing changes in the material’s surface during dynamic processes such as catalysis, electrochemistry, and corrosion. In situ and operando SIMS requires a controlled sample environment that mimics the conditions of interest. This may involve maintaining specific temperature, pressure, or gas atmospheres. The ability to perform SIMS under these controlled conditions is crucial for understanding how surface composition evolves during different processes. Microfluidic liquid cells, such as vacuum-compatible microfluidic reactor systems for analysis at the liquid vacuum interface (SALVI), were developed to be applied in in situ, in vivo, and in operando imaging of liquid surface as well as the air–liquid, liquid–liquid, and solid–liquid interfaces in the past decade [53,54,55]. SALVI is one of the first microfluidics-based reactors that enabled direct analysis of real-time changes in the material’s surface by combining ToF-SIMS [56]. Figure 2 shows an example that in situ/operando ToF-SIMS with SALVI is used to study the air- and pressure-sensitive green rust (GR) nanocrystalline synthesis at the molecular level.

In situ and operando SIMS represents a paradigm shift in surface analysis, allowing researchers to probe material surfaces in real time during dynamic processes. The principles underlying this technique encompass controlled sample environments, dynamic sampling, high temporal and spatial resolution, advanced mass spectrometry, and sophisticated data analysis. As technology continues to advance, in situ/operando SIMS is expected to play an increasingly vital role in unraveling the complexities of surface reactions and guiding the development of new materials and technologies. Nowadays, in situ and operando SIMS find applications in various fields, including catalysis, battery research, corrosion studies, and semiconductor device characterization. The ability to analyze surfaces under working conditions provides unique insights that are crucial for optimizing material performance and understanding fundamental processes.

### 3.3. SIMS Measurement Modality

There are three basic measurement modalities of SIMS: spectral analysis, imaging, and depth profiling. Among these, spectral analysis is the most straightforward and commonly employed method, which can acquire high-resolution mass spectra for the targeted surface. Throughout the mass spectra, spatial coordinates for each irradiated pixel are meticulously recorded. From the obtained mass spectra, specific ions can be discerned and selected, enabling the generation of ion images or chemical ion maps that illustrate the distribution of these ions across the analyzed area (x, y). The size of the analytical area can be varied widely, ranging from as small as 5 μm^2^ to larger regions spanning several millimeters [57]. Mass spectral analysis has been used for a variety of purposes, including but not limited to assessing the oxidation of metal surfaces, identifying contaminants, determining relative abundances of elements or molecules, exploring molecular orientations on surfaces, and distinguishing between block and random copolymers [58,59,60,61,62]. In imaging mode, the primary ion beam is precisely focused to augment lateral resolution. The beam is systematically rastered across the surface, resulting in the creation of a mass spectral ‘image’. This imaging approach proves invaluable for characterizing chemically distinct regions on the surface, providing a comprehensive understanding of the spatial distribution of various ions and molecular species.

For many systems, it is desirable to gain an understanding of how composition varies with depth, for example, the distribution of elements from the surface to the inner of a bronze lion [63]. Facilitating this exploration is the integration of an additional ion source, colloquially referred to as a sputter ion beam or gun. This adjunctive apparatus enables the implementation of depth profiling techniques, a vital approach for scrutinizing mass spectra in relation to varying depths within a sample. Presently, depth profiling is the predominant analytical mode. Utilizing fast surface erosion techniques, variations in elemental composition are methodically probed as a function of depth. The interface structure and the diffusion between layered structures can be meticulously examined with sub-nanometer depth resolution by employing low-impact energies. Also, by combining SI mapping with depth profiling, a 3D analysis can be obtained.

However, when conducting SIMS analysis, the intensity of SIs is contingent not only upon the concentration of the target element but also on the nature and composition of the material. This is commonly called the ‘matrix effect’, and it prevents SIMS from being directly quantitative [25,64,65]. The matrix effect manifests due to disparities in ionization rates and sputter yields across different materials. The first is a purely theoretical approach, wherein concentrations of various elements are directly calculated from ion counting rates. The second is an empirical approach, whereby concentrations are determined through the application of relative sensitivity factors (RSF) [66]. The RSFs determined from pure metals are generally used for the quantification of alloy materials. However, it is crucial to consider matrix effects arising from factors such as atomic density, electron attenuation lengths, and electron backscattering (in the case of Auger Electron Spectroscopy, AES) within the matrix materials [67]. For the accurate quantification of alloys, a recommended calibration method involves the use of reference materials. This approach minimizes matrix effects by employing pure element reference materials [68]. The ideal method for quantifying alloys entails utilizing a reference sample with the same composition, and the next best one is to use a calibration curve generated from reference samples with a series of different compositions spanning the unknown composition [68].

To date, the utilization of ion-implantation reference materials has proven highly effective in establishing the necessary standards, given its capability to encompass all elements and isotopes. The depth and concentration of the implant can be flexibly adjusted by modifying the implantation energy and dose, respectively. Ion-implanted samples have undergone analysis for nearly all non-radioactive elements. Notably, the adoption of this implantation technique has propelled SIMS from a semi-quantitative method to a technique that can provide intra-laboratory dose measurements with reproducibility of better than 1% relative standard deviation (RSD) for the magnetic sector, quadrupole, and ToF instruments. Quantification has been established for impurity and matrix species in a number of materials, such as Si_x_Ge_(1−x)_, Al_(x)_Ga_(1−x)_As, and Al_x_Ga_(1−x)_N [69,70,71,72].

When preparing a SIMS sample, it is important to maintain a clean surface to avoid the other factors that would influence measurement results. For example, one should use only polyethylene gloves because other gloves may contain silicones and introduce interferences. One should use only clean tools when handling samples. Sample preparation should be performed to remove hydrocarbon and silicone contaminants in lab space that is dedicated to SIMS use only. Materials need to be kept in a ‘clean’ environment before and after analysis. A laminar flow hood or a clean laboratory environment is strongly recommended for this purpose. During SIMS analysis, control samples are always needed, and the interference peaks from the substrate can be excluded when analyzing materials of interest. When conducting SIMS measurements, several different data points are usually acquired on one sample to ensure that measurements are representative of the whole material. The relative standard deviation percentage (RSD%) of peak area and height can be calculated to assess the reproducibility of the SIMS spectral measurements [73]. When the RSD% is below 5% in peak areas, the reproducibility of the SIMS spectral measurements is good [74]. Reasonable measurement reproducibility is the foundation of reliable SIMS analysis. When analyzing materials in SIMS, the primary ion beams are carefully selected depending on the nature and composition of the samples. Since SIMS provides semi-quantitative analysis, measurement precision or repeatability can be used to estimate measurement error [73,75]. Often, RSD% is used as a measure to indicate measurement precision and how consistent measurements are to offer data assurance.

## 4. SIMS Applications in Metals and Alloys

The continued development of SIMS instruments has led to the successful implementation of SIMS’s measurement modalities across many scientific disciplines due to their advantages in sensitivity and resolution. To help highlight the successful applications of SIMS as analytical tools, a number of case studies have been selected to demonstrate the advantages SIMS has in studying metals and alloys. These studies should be helpful for those unfamiliar with SIMS to categorize what work has been completed and what might be of interest for further consideration in their specific field of study. They are grouped by the type of SIMS utilized to best illustrate the strengths of each technique and then further separated into common applications for that specific technique. A section highlighting correlative imaging and complimentary techniques for SIMS is given to note an increasing trend in the use of SIMS in correlative metal analysis. The fields of study of the collected references, along with the employed instrument’s make and model, are summarized in Table 3.

### 4.1. ToF-SIMS

ToF-SIMS is an ideal choice for material analysis due to its parallel ion detection, high transmission, and high mass resolution. Therefore, fields that call for understanding small-scale interactions to comprehend behaviors, such as corrosion behavior, film characterization, and biomedical alloy, are all excellent examples of understanding what ToF-SIMS can perform for material analysis.

#### 4.1.1. Corrosion Behavior 

Localized corrosion is known across many fields, specifically as a cause of failure for metal and alloyed components. With the atomic scale interactions that guide corrosion behavior, SIMS, as a highly sensitive technique, allows for insight into both the in-depth chemical structure and elemental distribution within samples to determine the effects of environment and additives on corrosion behavior.

**Figure 3:**
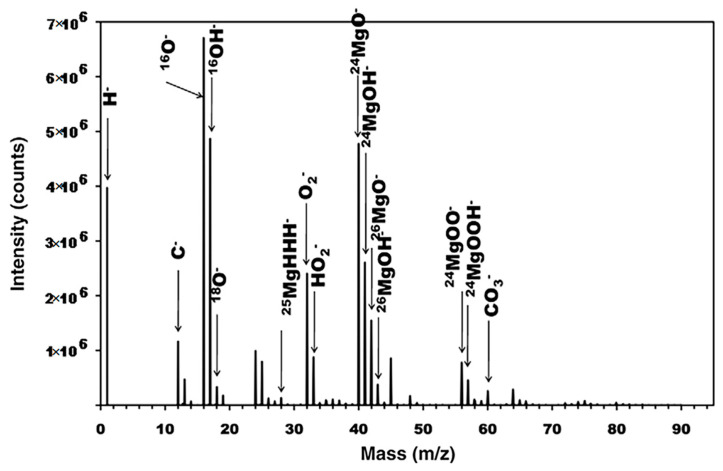
ToF-SIMS Spectrum of pure Mg after submersion in pure water. Reproduced with permission from Ref. [78]. Copyright 2009 Elsevier.

For instance, Li et al. applied ToF-SIMS 3D imaging to investigate granular corrosion as a precursor to stress corrosion cracking for Al–Cu–Li alloys [76]. SIMS’s sensitivity allowed for the analysis of sub-ppm to ppb levels of lighter mass elemental distributions, such as Li. Al–Cu–Fe–Mn intermetallic particles were shown to be preferential sites for corrosion in Al alloys. Two-dimensional (2D) images showed higher intensities of alloying elements Cu, Fe, and Mn and lower intensities of Al within the boundaries of the intermetallic particle region of interest. Lighter mass elements were observed to not be present within the intermetallic particles where localized corrosion occurred. In another study, Esmaily et al. demonstrated ToF-SIMS’s surface sensitivity to study the corrosion mechanisms of Mg alloys at sub-zero temperatures [77]. Two-dimensional imaging of selected ionic species, including Cl^−^ and AlO^−^, demonstrated that the redistribution of light atoms was temperature-dependent. At higher temperatures, AlO^−^ was found a distance away from the anodic sites, and it had not migrated toward the cathodic sites at sub-zero temperatures. Seyeux et al. have shown how ToF-SIMS can provide insight into corroded layers or anti-corrosion films upon engineered surfaces [78]. SIMS spectral analysis and depth profiling were performed to investigate the presence of MgH_2_ and confirm its formation in submerged Mg. SIMS’s high sensitivity makes it ideal for validation of this process. In a previous study, MgH_2_ formation was identified from weak XRD data on submerged pure Mg [79]. Spectral analysis revealed the presence of both MgOH^−^ and MgO^−^, as well as MgH_2_, which can be seen in dept profiling (Figure 3), indicating that the surface layer was split into a hydroxide dominated outer layer and oxide dominated inner layer. MgH_2_ was at a much lower intensity than either the oxide or hydroxide signals and decreased from the film’s surface, which hinted that further work could illustrate its possible role in corrosion mechanics.

**Table 3:** Summary of representative metals and alloys analysis using SIMS.

Type	Field of Study	Instrument Make and Model	Metallic Ions Studied	References
ToF-SIMS	Corrosion Behavior	IONTOF V	Al, Cu, Li, Ga, Fe, Mn, Mg Zn, Pb, Sn, Y	[76,77,79,80,81,82]
	Thin Film/Oxide Properties	IONTOF IV, V	Cr, Ni, Cu, Al, Li, V, Ag Ti	[78,83,84,85,86,87,88,89]
	Biomedical Implants	IONTOF V, Physical Electronics PHI 7200	Fe, Cr, Ti, Nb, Ca	[90,91,92,93,94]
Magnetic SIMS	Semiconductor Analysis	CAMECA IMS-6F, CAMECA IMS-4FE7 CAMECA IMS-4f	Hf, Cu, In, Ga	[95,96,97,98,99,100]
	Additive Transport	CAMECA IMS-6F, CAMECA IMS 5F, DTDC SHRIMP	Al, Li, Cu, Y, Mn, Zn, Mg, Zr, Ga, Fe, Cr, Ni, U	[101,102,103,104,105,106]
	Geologic Analysis	CAMECA IMS-6F, DTDC SHRIMP	U, Pb, Hf, Lu	[106,107,108,109,110]
	Cosmochemical Analysis	CAMECA IMS 1280, CAMECA IMS 1270 DTDC SHRIMP	Pb, Fe, Ni, Mn, Cr	[111,112,113,114,115]
	Nuclear Safeguard Analysis	CAMECA IMS 1280, CAMECA IMS 1280-HR	U	[116,117,118,119,120]
NanoSIMS	Segregation Analysis	CAMECA NanoSIMS 50L, CAMECA NanoSIMS 50	Mo, Cr, Fe, Nb, Ti, Al, Mg, Ni	[98,121,122,123,124,125,126]
	Hydrogen Isotopic Analysis	CAMECA NanoSIMS 50	Mn, Cr, Mo, Ni, Fe	[127,128,129,130,131,132]

**Figure 4:**
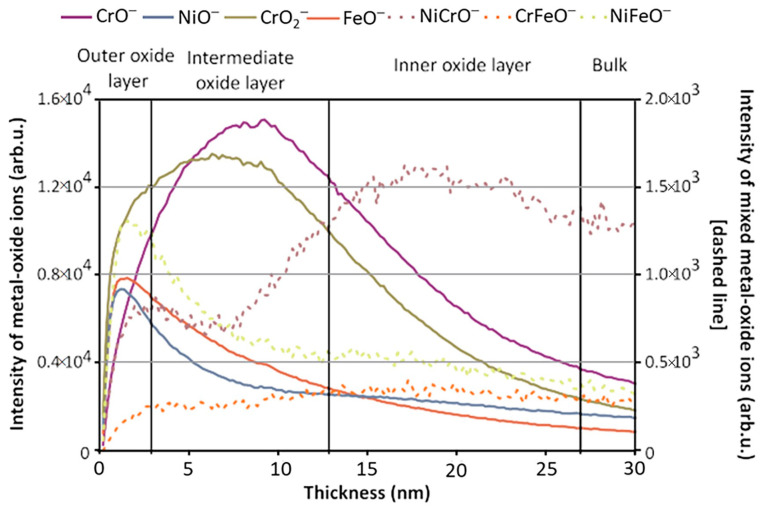
ToF-SIMS depth profile (negative mode) of an SG tube (alloy 690) thermally treated in air for 4 h at 500 °C (Cs^+^ 0.5 keV, current 30 nA). Reproduced with permission from Ref. [133]. Copyright 2012 Wiley.

ToF-SIMS’s ability to discern both organic and inorganic species via depth profiling and imaging can facilitate the characterization of metals and alloys, a valuable tool for the analysis of cultural artifacts. Yin et al. analyzed a historical copper alloy, which contained Cu, Zn, Tn, and Pb, from a bronze lion and an official Seal from the Han dynasty [63]. The cause of the artifact’s anticorrosive properties was dissected using static and dynamic SIMS to obtain surface composition and depth-resolved information, respectively, to observe the method the artisans utilized in its manufacture. The depth profile result showed that Ni enrichment was discovered at the surface of the artifact, which was mirrored across three of the four locations where analysis was performed. Mazenc et al. studied the behavior of thermally oxidized films formed on nickel-based 690 alloys in high-temperature water [133]. The depth profile of a steam generator (SG) tube (alloy 690) was sputtered at 0.5 keV (30 nA), with key results shown in Figure 4. The oxide layer can be discerned into three distinct parts. The outer layer is comprised of a mixed oxide layer rich in Ni and Fe, and the intermediate layer is predominantly composed of chromium oxide. The inner layer, marked by a pronounced NiCrO^−^ signal, corresponds to a spinel-rich NiCr_2_O_4_ portion. Trace elements such as gold and lead were also observed thanks to SIMS’s high sensitivity. Lead was seen to disrupt the distribution of major elements and a Ni/Zn alloy, which explained the anti-corrosion behavior exhibited by the artifact [134].

#### 4.1.2. Thin Films and Oxide Layers

Oxides and other surface layers play key roles in nuclear and material science, from the engineering of material properties to environmental remediation in materials from steel to paint mediums [135,136]. The characterization of these films and layers’ structures is therefore important in understanding the properties that they impart upon their host material.

The loss of Cr from stainless steels and Ni-base alloys has been reported to be a cause of significant loss of performance, as well as a poison of other elements of the work environment, such as CrO_3_ poisoning solid-state battery cathodes [87]. Thus, the growth and transport mechanism of chromium oxide layers is of immense importance. Poulain et al. investigated the oxidation of chromium at 300 °C to observe and determine the governing transport behavior within the oxide film at elevated temperatures. ToF-SIMS was used to analyze a polished sample to obtain spatially resolved depth profiles of the oxide layer. It was confirmed that oxygen diffused through the oxide layer to react with the metal at the oxide/metal interface to grow. The excellent mass resolution allowed for depth profiling to also reveal a second mechanism between ^16^O and ^18^O that takes place at the oxide surface as well as to separate the oxide into two regions. One layer is where Cr and ^18^O dominate, and an outer layer is where the ^18^O is exchanged for ^16^O, showing an inward diffusion of oxygen. 

SIMS’s capacity to study surface layers is useful in optimization in addition to characterization. Byrne et al. studied copper retention in a thin film of SiO_2_ [84]. Depth profiling was used to determine if the inclusion of Al to form a Cu–Al alloy would retain the Cu inside the film. The inward diffusion of Cu into the SiO_2_ layer for both a pure Cu and a Cu–Al alloy layer served to evaluate the role in evaluating aluminum’s addition to the metal film. It was discovered that diffusion into the SiO2 substrate occurred in the pure Cu sample, while in the Cu–Al alloy sample, the Cu was retained in the surface alloy layer. These behaviors demonstrated the benefit of doping Cu surface films with Al for use as stabilized dielectric device structures. Jolanta et al. have similarly relied on the elemental distributions of Li to observe its mobility in V oxide films [87]. In another study, positive and negative depth profiles were taken to measure the intercalation mechanism of Li for battery host material development. ToF-SIMS’s excellent sensitivity when measuring Li has been shown in studies of tungsten oxides [137]. The distribution of the Li was measured and showed a maximum presence in the outer V2O5 layer but was also found at the oxide/metal substrate, indicating Li diffusion across to the inner oxide layers below via grain boundaries. ToF-SIMS was also used to study copper adsorption on pyrite. It was found that Cu^2+^ ions could result in the activation of pyrite during separation in processing, thus lowering the grade of copper produced from the process [138]. SIMS surface sensitivity revealed that pyrite surfaces were activated with high and low Cu concentrations at neutral pH, with the surface dominated by Cu and Fe hydroxides. At low pH, Cu(OH)_2_ formed a layer on the surface of the pyrite.

ToF-SIMS was shown to be an effective tool in analyzing pigment and binder alteration processes in the paint layers of ‘Le Bonheur de vivre’ (1905–1906, The Barnes Foundation) by Henri Matisse due to its ability to image inorganic and organic elements at a µm spatial resolution [139]. Imaging of elemental and molecular signals related to CdS pigment, associated binding medium, and degradation products were taken from multiple locations across the painting and were chosen for analysis. These samples were compared to artificially aged reference paints to investigate the processes of pigment and binder degradation, along with previous restoration efforts. Results showed that SIMS allowed for the identification of degradation products previously unobserved with methods such as Scanning Electron Microscope coupled with Energy Dispersive X-ray (SEM-EDX). Across the four samples, CdS pigment and CdCl_2_ were adjoined throughout the various paint layers. The presence of CdSO_4_ and CdCO_3_ identified the mechanism through which the CdS pigment was degraded and explained the now faded color of the work. SIMS also identified CdC_2_O_4_ as evidence of the binding medium’s degradation, which was a potential source of the fragility of the upper paint layers present in the painting.

#### 4.1.3. Metals and Alloys for Biomedical Applications 

ToF-SIMS is commonly used to study biological samples, from microbes to drug analysis [4,140]. Biomedical surface preparation for implants, where residue can be harmful if not removed, has benefitted from SIMS’s ability to characterize surface interfaces where interfacing of implant and bone is desired.

Göttlicher et al. investigated low-temperature oxidation behaviors induced by plasma for orthopedic Ti-40Nb alloys [91]. Low-temperature oxidation was induced by plasma to study the growth of the surface oxide. ToF-SIMS depth profiles showed that the impurities SiO_2_, FeO, CrO, and Pt decreased in intensity with surface depth. Suggestions were made to prevent impurity transference, such as adding additional magnetic confinement of the plasma. Ti ions were also observed to have a faster migration than Nb, leading to concentration gradients after exposure to the plasma. Eriksson et al. focus on devices post-implantation [90]. Six implants, each with various surface treatments, were placed within the tibia of a rat for 7 days. They were then removed to compare the effect of porosity on hydroxyapatite formation, called mineralization, using ToF-SIMS imaging. Positive spectra were obtained, and the profiles of select ions were imaged. Ca^2+^ and CaOH, characteristic peaks of hydroxyapatite, were detected across all six surfaces after one week. From the results, porosity was shown to have a clear influence over mineralization. It was also concluded that for the one-week period, ToF-SIMS was more reliable for predicting biocompatibility than other markers such as bone-to-metal-contact.

In another study, Xu et al. considered osteoconduction of alkaline-treated Ti surfaces of two-month implants to investigate the chemical composition of new bone formed on the treated surface [92]. ToF-SIMS analysis indicated changes in the bone/Ti interface and uniform distribution of Ca and P deficiency. Ca was shown to have a higher deposition rate on the treated surface, with the SIMS imaging providing further insight into bone mineralization. The analysis also indicated lower concentrations of PO_4_ and OH, constituents of hydroxyapatite, near the material interface. This deficiency implies an intermediate mineral phase or hydroxyapatite with large numbers of imperfections.

### 4.2. Magnetic SIMS

Due to the continually operating ion beam, magnetic SIMS offers depth profiling capabilities with extremely high depth resolution. This, coupled with high SI transmission and a high mass resolving power, makes magnetic sector SIMS an excellent option when extremely precise elemental and isotopic analysis is required. Semiconductor analysis, alloy-element distribution, and geologic dating are all excellent applications for magnetic-sector SIMS due to their features of precise examination of often small quantities of analyte within a given substance.

#### 4.2.1. Semiconductor Materials

Magnetic-sector SIMS is an extremely useful tool for characterizing semiconductor materials. Depth profiles and imaging offer data into diffusion barriers or dopants, and elemental distribution can track the evolution of changing microstructural elements and elemental additives. Gu et al. have shown how SIMS may be used to study dielectrics such as HfSixOy, which is one of the most promising high-k materials. High-k materials are a class that is under study in an attempt to reduce semiconductor device dimensions [95]. One requirement for this type of dielectric is that the constituent elements cannot be allowed to diffuse into adjacent regions in the device during processing. SIMS analysis provides results that were stated to be unobtainable by any other method used previously. Sufficient depth resolution was obtained to define the substrate from the HfSiO layer, as well as the identification of an apparent interfacial layer that was previously thought to be SiO_2_ but was shown to contain Hf as well, with no indication of Hf diffusion into the substrate observed.

Quantification of data using SIMS is possible, as is shown by Lee et al. in studying Cu(In,Ga)Se_2_ (CIGS) thin films [141]. CIGS films are popular due to their high absorption coefficients and band gap properties. Analysis of these absorbing layers, therefore, allows for insight into increasing the efficiency of solar cells that contain CIGS. Magnetic-sector SIMS depth profiling of copper, indium, gallium, and selenium was performed and showed a selenium-rich, copper-poor surface region in the CIGS film. Relative sensitivity factors were calculated using integrated intensities gathered from SIMS data. Atomic compositions calculated were shown to be within 1% of other quantification methods, such as inductively coupled plasma atomic emission spectroscopy. This method was found to have great advantages in quantification, and magnetic SIMS was demonstrated for the potential application of magnetic-SIMS for the quantification of these multilayer thin films. 

#### 4.2.2. Small Additive Transport and Incorporation in Materials

Given the ever-increasing complexity of alloy compositions, analysis of the behavior of small percentages of alloying elements is paramount. Castro et al. apply magnetic-sector SIMS imaging to an Al–Li alloy to observe small weight percent additions of Cu, Mn, Zn, and Mg, which are commonly added to obtain certain desirable properties and are usually added into the microstructure as fine nanoscale precipitates. Other techniques, such as EDX, have insufficient spatial resolution or sensitivity for such tasks [102]. Magnetic sector SIMS was employed for this purpose to observe the microstructural distribution of these low-percent additives. Images of ^7^Li, ^55^Mn, ^56^Fe, and ^63^Cu were acquired for analysis, where Li was shown to segregate at grain boundaries in a phase identified as the phase (Al_2_CuLi, T1) based on the work by Xu et al. [126]. Magnesium was also detected despite a relatively low concentration of 0.2%. It was concluded that the alloy was not homogenous, comprising precipitate phases with Mg partitioning and Zn incorporation into an interface phase. Overall, the resolution of the magnetic sector instrument helped to identify these nanodomains where other techniques, such as EDX and nano-XRF, could not [142,143].

III-Nitride (i.e., GaN, AlGaN) is of interest in high-power electronic and optoelectronic devices, where quantification of impurity species allows for information relating to dopant and impurity control. Gu et al. analyzed such materials with magnetic-sector SIMS, relying on its high dynamic depth resolution and high transmission, obtaining relative sensitivity factors for impurity species [104]. These factors were calculated for various impurities in AlGaN, which revealed that the relative sensitivity factors for Mg and Si appeared to remain relatively stable when normalized to N-containing matrix ions. This normalization was stated to provide a valuable quantitative tool for analyzing such materials due to the constant concentration of matrix ions in AlGaN.

Titanite, a titanium silicate mineral, can be grown with trace metallic inclusions for use as standards or as experimental starting points for other analytical techniques. Mazdab et al. use the SHRIMP instrument to study trace element incorporation in natural titanite [106]. Sc, Cr, Ni, Y, Zr, Nb, Hf, Ta, Th, and U can all be identified as trace elements of approximately 50 ppm in natural titanite as well as when doped in grown titanite crystals. Grown titanite was shown to also contain Na and smaller concentrations of B from the flux used in manufacturing. SHRIMP analysis confirmed that the trace elements were successfully incorporated into structural sites.

#### 4.2.3. Geologic Formations and Minerals 

One large application of magnetic SIMS, particularly the Sensitive High-Resolution Ion Microprobe (SHRIMP) instrument, is the geosciences, being well suited for analysis of geological formations, using ionic ratios to date minerals and rock formations. Many studies, including one by Zhuchenko et al., focus on the analysis and dating of zircon, a mineral that holds information from ancient geological eras [108]. Zhuchenko employs a SHRIMP instrument to date zircons by analyzing the ratio of U-Pb ions to gain insight into ancient geological activity. The analyzed zircons were taken from mafic granulite, and the zircon’s U–Pb ratio dating revealed the rough timeline of the beginning of magmatism in the region of interest (Ukraine), as well as another, newer recrystallization period that corresponded to a separate metamorphic era. Zirconium dating was also applied to better understand the synchroneity of geological activities across what is now Asia and North America [107]. The analysis revealed the age of the zircons collected from the area of interest, the upper Xieshuihe formation, in south China, fell within the error margins for SHRIMP analysis of zircons originating from separate geological formations in northern Idaho and Utah, USA, as well as in Yukon Canada. This analysis suggested that the two differing locations were subjected to similar conditions around similar times, dating the geological behavior due to the formation conditions and, thus, isotopic distributions of the analyzed zircons.

Shatkov et al. used a similar method to analyze zircons in the uranium-bearing Transbaikalia geological structure in Russia to determine the formation of the Tulukuev caldera [144]. U-Pb SHRIMP analysis revealed lower counts of U and Th than other regions of the caldera, mostly the core and close edges, than the further, cooler regions. SHRIMP analysis also showed that locations of radioactive uranium isotopes were restricted to certain regions as well, which was evidence of U being moved and separated throughout the structure, hinting at the formation of the structure. Shi et al. employed SHRIMP to analyze Hf isotopes alongside U–Pb analysis. Samples were taken from various types of rock from the North China Craton. SHRIMP zircon ages for each type documented granitoid formation and allowed for an understanding of the date of underlying formative metamorphic events, including evidence of vertical crust growth based on isotopic Hf ratios with Lu varying from region to region within the area of interest.

SIMS and Fourier Transform Infrared (FTIR) analysis has been used to measure hydrogen abundance within both experimentally annealed natural mantle materials [145]. The abundance was employed for calibration to be used in measuring H_2_O concentrations in a variety of minerals. The relationship between anhydrous materials and silicate melts was selected to aid in understanding the distribution of H between various phases of the mantle and the processes that influence H distribution. FTIR has had previous success in measuring low abundances of hydrogen in small samples. SIMS offered considerable advantages for quantitative analysis of hydrogen equilibrated between phases, including insensitivity to crystal orientation, low detection limit, and high spatial resolution. FTIR and SIMS were compared for their capacity to analyze anhydrous materials through annealing experiments using synthesized crystals with varying H_2_O concentrations in the testing environment. Results showed that SIMS had better lateral and depth resolution than FTIR, with FTIR averaged absorption over the full thickness of the sample. Using both techniques, it was possible to compare the minimum atom count required for each technique, with SIMS requiring three orders of magnitude fewer atoms than FTIR and thus being three orders of magnitude more sensitive for measuring hydrogen abundance. SIMS and FTIR could be employed as complimentary techniques. SIMS allows for in situ analysis of small single spots, and FTIR provides information on substitution mechanisms.

This notion of SIMS’s superiority over FTIR, but having the potential to be complementary, was shared when volatile species in volcanic glasses were measured by Hauri et al. [146]. SIMS was utilized to measure the isotopic abundances and compositions of volatile elements in standard glasses and compared to FTIR measurements of the same materials for use in studying volcanic degassing of volatile species. The comparison showed that SIMS offered easier sample preparation, sufficient detection limits, and sufficient spatial resolution to collect data from secondary phases, which hindered other vacuum extraction techniques [147]. Its excellent resolution also facilitated the acquisition of isotopic data for H, C, and S, which aided in providing constraints to magma evolutions, degassing, and contamination processes.

### 4.3. Large Geometry SIMS

An important role for SIMS is the characterization of particles. Large geometry SIMS achieves high transmission at high mass resolving power and is commonly utilized to study environmental samples, such as meteorites, and as nuclear safeguards by measuring actinide particle compositions.

#### 4.3.1. Extraterrestrial Materials 

Large geometry SIMS provides an optimal platform for cosmochemistry due to the capacity for in-situ trace element analysis of complex minerals. In applying large geometry SIMS to the study of extraterrestrial materials, Merle et al. employed large geometry SIMS to study lunar basalt and mafic plutonic rocks [113]. Their objective was to further understand the early moon’s crust-mantle differentiation by obtaining radiogenic isotopic compositions due to the constraints they place upon the composition of the mantle source they formed from. The accurate measuring of the selected Pb ions for dating, as well as the excellent spatial resolution achieved by the instrument, allowed for precise comparison of Pb isotopic ratios in samples. Dating results showed clear evidence of magmatic activity on the moon from 3100 to 300 million years ago, and it was not continuous as was previously suggested. Pack et al. used large geometry SIMS to investigate the silicon content of iron meteorites, which are predominantly made of iron and nickel alloys, as well as minor inclusions of other compounds of FeS, FeNi_3_C, and silicates [114]. The meteorites chosen are of a type with largely unfractionalized trace elements [148]. This analysis was performed due to the thermal history of the meteorites being derived from the metal structure and phases present within the bulk. Thus, if a conventional theory and model of formation are correct, then silicates and metals must have entered an equilibrium phase. Silicate partitioning into the metal phase can be calculated and compared to the stability and activity coefficients of Si in Ni and Fe alloys. This comparison allowed for silicate concentrations to be calculated and compared with the Si contents measured in the meteorites. ^28^SI and ^54^Fe were utilized to represent Si and Fe content, and sensitivity factors for ^28^Si/^54^Fe were determined across 15 meteorite samples. No silicate inclusions were observed, with Si content low and steady across all samples within a narrow range. Ni-rich regions were identified as probable intergrowths of metallic phases, and it was suggested that the observed low Si presence was due to metal and silicates achieving solid state equilibrium below 1270 K. 

Soens et al. utilized a similar experimental apparatus to analyze a refractory phase-bearing micrometeorite to understand its origin [115]. It was found to be rich in silicates as well as Ca-Al inclusions and Mg oxides. Oxygen concentrations were found to be consistent with the other meteorites of its type. Based on these inclusions of MgO, Ca, and Al, as well as the measured oxygen isotope concentrations, it was suggested that its origin might be linked to main belt or Jupiter family comets, based on similarities of previous results.

#### 4.3.2. Particle Heterogeneity/Homogeneity 

Jovanovic et al. summarize three different large geometry instruments analyzing two uranium dioxide pellets with similar bulk isotope compositions but different spatial uranium isotope distributions [117]. Each instrument analyzed a set of pellets and obtained and reported results in terms of isotopic ratios and distributions. They demonstrated the capacity for measurement reproducibility for each instrument produced similar results across the two samples. The first particle, it was concluded, was a combination of low-enriched uranium and depleted uranium. SIMS was the only technique among several used to detect and characterize the 235U/238U ratio of the second particle due to that particle’s smaller domains, and it was found to be similar in composition to the first. Large geometry SIMS was able to perform high precision and high spatial resolution characterization for the two particles and provide direct spatial visualization and structural information on isotope distribution.

Varga et al. would perform a similar analysis using large geometry SIMS to verify the analysis of inhomogeneous samples containing uranium particles of various enrichments [120]. The measured uranium standards were U_3_O_8_ with given U^234^/U^238^ and U^235^/U^238^ ratios and were of various sizes ranging from sub-micrometers to a few hundred micrometers. Around 2200 uranium particles were found in automatic screening. More precise measurements found around 30 particles that were able to be separated into two different populations based on their ^235^U enrichment values of 1.01% and 3.06%, respectively. It was noted that the two different reference samples demonstrated differences between these populations. One had two statistically significant ^235^U populations present, one at 0.97% and the other at 1.01%, while the other reference sample was found to be more homogenous. Overall, a similar conclusion was drawn, as in Jovanovic et al.’s study, that large geometry SIMS could be used to validate and confirm uranium ratios, providing an excellent tool for nuclear safeguarding. 

It has quickly expanded in biological and medical research domains and has been applied in diverse fields, including material sciences, cosmochemistry, and geosciences.

### 4.4. NanoSIMS

NanoSIMS has high spatial resolution and high collection efficiency, which allows for specialization in elemental and isotopic analysis. NanoSIMS, therefore, is an ideal technique for use in fields from analyzing biological samples, such as single cells, clays, and sediments, geochemistry, cosmochemistry, and material science, where it is commonly applied for both analyzing nanoscale elemental segregation for light element and isotopic analysis. 

#### 4.4.1. Study of Elemental Segregation in Metals and Alloys Using NanoSIMS

Elemental segregation at grain boundaries is a common cause of altered behavioral properties such as embrittlement [149]. A study of Inconel 718 by Talukder et al. focuses on small additions of B, P, and C, which have been shown to result in drastic changes in mechanical behavior [121]. While the alloy is very desirable for its mechanical properties, its preferred method of repair is welding. B and P additions improve the life of the alloy but have been shown to have detrimental effects on weldability, while C helps to mitigate this negative effect of the B and P additions. NanoSIMS is used to observe the presence of B, P, and C segregations to the grain boundaries of the alloy, as the alloy suffers micro-fissuring during welding in the heat-affected zone near the welding site. Carbon’s effect in mitigating the negative welding ability was investigated. Two alloy compositions—one with 0.006 wt.% of added C content and one with 0.033 wt.% C content—were selected for study. Selected ions for analysis included ^12^C^−^, ^16^O^−^, ^11^B, ^16^O^−^, ^31^P^−^, and ^58^Ni^−^.

Images to accompany the elemental analysis were acquired. The images and elemental analysis show that the Ni content was identical in both measured alloys. B segregation was observed similarly for both alloy samples, although it was significantly reduced for the sample with the increased carbon presence. Alternatively, no P or C segregation could be detected for the grain boundary, but the author stated that it was possibly due to the segregation of these elements being below the detection limit for the given experimental conditions. The B to Ni ratio across the grain boundary shows a dramatic increase in the B segregation in the sample with higher carbon content. Therefore, it is hypothesized that carbon influences the segregation of B across a larger area away from the material’s grain boundaries than was originally thought. Rosa et al. also investigated B inclusion but focused on its segregation at austenite grain boundaries in low-carbon steel [122]. NanoSIMS was selected based on the expected concentrations of B at grain boundaries because other techniques, such as atom probe tomography (APT), had insufficient detection limits (a few ppm for NanoSIMS vs. tens of ppm for APT) [8,150]. The segregation of B at the grain boundaries as a function of temperature was determined using NanoSIMS, and it revealed boron distribution in the microstructure consisting of martensite. Although various boundaries were observed, B was only found at observed γGBs and not at formed packets, blocks, and laths. This NanoSIMS data contributed to the construction of modeling boron segregation kinetics. The modeling and experimental observations revealed that the B inclusions were very mobile at high temperatures, with fast quenching being insufficient to restrain segregation behavior during the quenching process. It was also observed that the ratio between boron segregation at γGBs and boron in solution in grains decreases with increasing temperature. 

**Figure 5:**
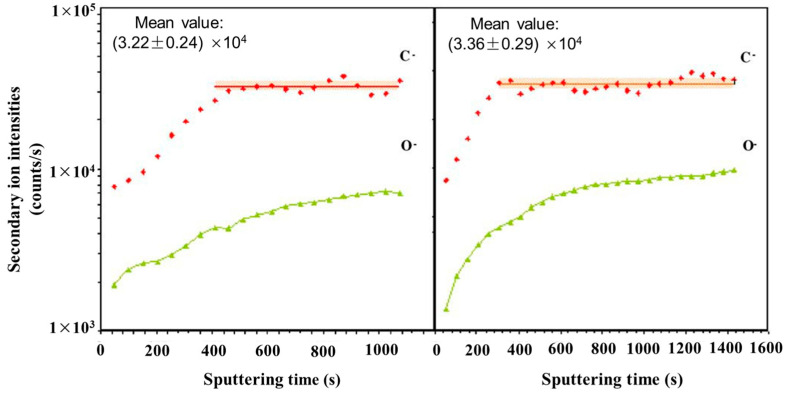
Evolution of carbon and oxygen secondary ion intensities versus sputtering time during two analyses in a sample with 0.97 at.% C. Adapted from Ref. [125].

A multi-phase steel was examined using NanoSIMS to study the microstructural distribution of carbon [125]. NanoSIMS was selected due to its up to 50 nm lateral resolution and high sensitivity, allowing for small changes in carbon content to be observed. The techniques used previously, such as Transmission Electron Microscopy using parallel electron energy loss spectroscopy (TEM-PEELS), are very complex, and thus NanoSIMS provided an alternative method utilizing its excellent lateral resolution and high sensitivity. The carbon concentrations ranged from 0.2 wt.% to 0.8 wt.%. With a 100 nm probe, the sensitivity of the technique is sufficient to detect small variations of carbon within the same phase. Figure 5 shows the carbon content of a bainite/martensite sample vs. sputtering time. Carbon was shown to have been enriched in the martensite regions due to bainite transformation. High carbon content was also shown in areas perpendicular to ferrite laths.

When compared with other microanalysis techniques, NanoSIMS had the ability to study the same area as in SEM and, if offered, visualization of carbon repartition within the microstructure of the steel. It was also concluded that because the detection limit was so low, 0.0063 wt.% for carbon in iron, the characterization of non-stable phases, such as bainite, which contained low concentrations of carbon, was possible. Nano SIMS allowed for a more complete understanding of bainitic transformation where the lack of quality data was at least partly assigned to the lack of detectability for techniques such as SEM and the higher detection limit of techniques, such as TEM-PEELS (1000 ppm).

#### 4.4.2. Hydrogen Isotopes in Metals and Alloys

**Figure 6:**
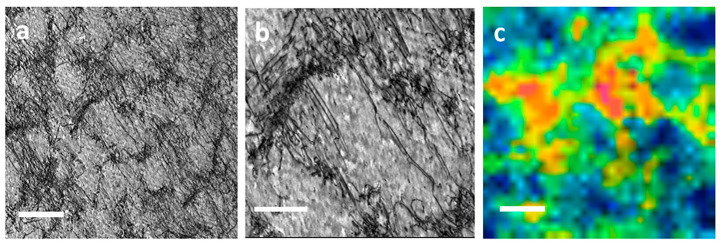
Dislocation structure at the crack tip. (**a**) TEM bright field image approximately 4–5 μm from crack. Scale bar is 500 nm; (**b**) Same region at higher magnification showing dislocations clusters observed in a. Scale bar is 200 nm; (**c**) 2H/16O ratio displayed as HSI image in the crack wake region 4–5 μm from the crack. The scale bar is 500 nm. Reproduced with permission from Ref. [130]. Copyright 2018 npj.

NanoSIMS offers the ability to analyze hydrogen isotopes such as deuterium and tritium, and it has an advantage when studying behaviors such as hydrogen embrittlement and blistering. Greg McMahon investigated the role of hydrogens in the deterioration of materials, specifically structural materials, through hydrogen-assisted cracking [130]. APT was also previously utilized as a possible option due to its good atomic resolution, but it has a much smaller sample volume (100,000 nm^3^ vs. 1 × 10^9^ nm^3^ S) and higher detection limits when compared to NanoSIMS or SIMS [8,130]. The latter is generally ten times better in detection sensitivity. The same is true with scanning probe methods such as scanning electrochemical microscope (SECM) analysis in which quantification is possible but whose resolution is on the order of hundreds of microns. NanoSIMS imaging was considered a prime technique to bridge these two extremes of operating parameters, providing better resolution than, for example, tritium audiography and better sample volume and detection sensitivity than APT. McMahon shows the distribution of hydrogen in the form of deuterium around primary and tertiary crack fatigue crack tips in two stainless steels. Deuterium, with its low natural abundance, was chosen for analysis. The ratio of deuterium to oxygen was determined due to oxygen’s presence as a matrix signal, with images acquired approximately 4–5 μm ahead of the crack tips. A cellular structure was found and is present in Figure 6, where dislocations create the structure’s bounds (Figure 6b). Bringing Figure 6a,b to even further magnifications allows for a hue saturation intensity image for deuterium and oxygen. These images revealed localized regions with clusters of enriched D/O ratio values and suggest that deuterium is trapped at sites of dislocations sinks. It was described that the distribution of these deuterium hot spots in localized regions might point to hydrogen influencing the steel’s deformation process and help narrow down which of the specific hydrogen-assisted cracking theorems are responsible for crack growth. This result was stated to agree with recent modeling efforts by Dadfarnia et al. and offers a way to validate models of hydrogen transport by dislocations using NanoSIMS [151].

Tarzimoghadam et al. investigated the hydrogen distribution and desorption behavior of a Ni-Nb alloy using NanoSIMS. The effect of the needle δ phase on hydrogen embrittlement was studied by mapping the hydrogen distribution within the Ni–Nb alloy. Applications of this alloy include use in hydrogen-containing atmospheres, which required investigation into the hydrogen distribution and embrittlement behavior of the alloy, to which NanoSIMS’s excellent resolution makes it a logical choice for analysis. Previous studies revealed that the δ phase affects the alloy’s sensitivity to hydrogen embrittlement; thus, the relationship between this phase and hydrogen trapping is of great interest. NanoSIMS analysis enabled the deuterium distribution within the microstructure to be detected and mapped. The results confirmed higher deuterium content in the Ni–Nb solid solution than in the δ phase.

In pressurized water reactors, the cladding around fuel rods is often made with zirconium alloy tubes, chosen for their low neutron capture cross section and good oxidation resistance. Therefore, understanding the hydrogen pickup of these at operating temperatures allows for the safer operation of these reactors. Li et al. described a method of 3D mapping deuterium distribution in oxidized Zircaloy-4 [128]. The high resolution required for the mapping of deuterium made NanoSIMS an excellent technique. Comparison among cross-sectional and depth profile measurements of the alloy showed the 3D distribution of deuterium in the material with the morphology of the deuterium trapping sites suggested. It was shown that the deuterium concentrated in the oxide near the water/oxide interface. A gradual decrease in deuterium concentrations was observed when approaching the oxide/metal interface. This behavior was interrupted by the local trapping sites (i.e., porosity, cracks) that were linked by diffusion paths into the metal bulk.

In another study, NanoSIMS was used to determine hydrogen’s distribution through the Zr oxide growth of Zircaloy-4, and various ratios of a Zr–Nb alloy were obtained [129]. Subjecting the materials to neutron irradiation was shown to increase the deuterium diffusion coefficient, the deuterium concentration trapped within the oxide, as well as the pickup fraction. Results, similar to Li et al., showed a concentration of decreasing deuterium from the oxide/water interface towards the deeper oxide layers with strong upticks in deuterium concentration hinting at strong trapping sites within the oxide. Zircaloy samples were found to have a high deuterium trapping ratio in the oxide layer and a high diffusion coefficient in the oxides. The diffusion coefficient in Nb containing samples’ oxides was much lower, a repeated result for similar conditions and materials from previous studies. NanoSIMS has been successfully applied for the hydrogen-induced cracking behavior of a Ni-based alloy [127]. NanoSIMS imaging produced ion and ratio maps taken from the passive oxide layer. Deuterium enrichments were found along dislocation slip bands as well as the intersections between them. This observation was attributed to hydrogen diffusion through mobile dislocations. Deuterium was also observed in twin boundary enrichments and along a particular phase boundary that is exhibited within the studied alloy.

### 4.5. Correlative Imaging Using SIMS

SIMS’s unique benefits can allow it to complement a wide variety of analysis techniques. Correlative imaging allows for a diverse range of information to benefit and improve upon the strengths of other techniques beyond what could be performed using isolated methods. The study of irradiated materials and semiconductors is characterized by complex microstructural evolution and is a common application of SIMS correlative imaging techniques. 

#### 4.5.1. Irradiated Materials 

**Figure 7:**
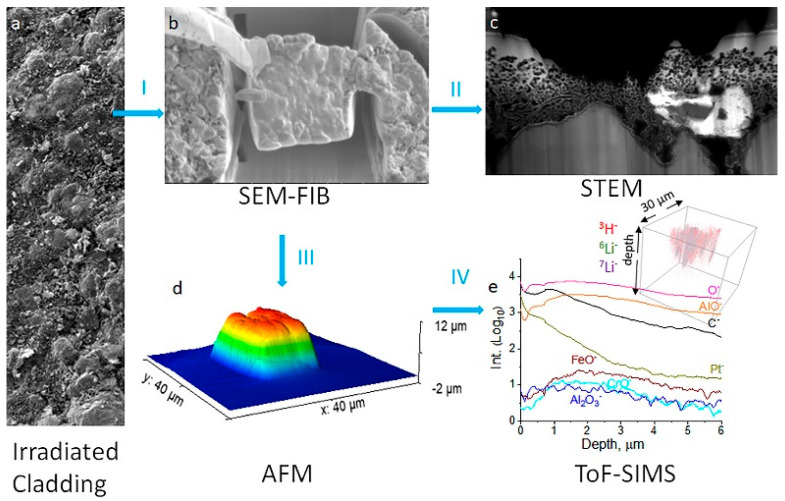
Multimodal chemical imaging of an irradiated tube (**a**) using (**b**) SEM-FIB to prepare the lift-out, (**c**) STEM to determine nanostructures and elemental mapping, (**d**) AFM to obtain the lift-out dimensions nondestructively, and (**e**) ToF-SIMS to acquire sensitive surface and isotopic, elemental, and molecular 3D mapping. Rainbow color indicates sample topographical height. Blue arrows are used to show workflow. Reproduced with permission from Ref. [88]. Copyright 2021 Elsevier.

Tritium and Li (^6^Li and ^7^Li) transport within neutron-irradiated functional intermetallic coatings, specifically Fe–Al alloys, are common concerns for their use in fission and future nuclear fusion applications [88]. Yu et al. used a focused ion beam with scanning electron microscopes (FIB-SEM) to prepare lift-outs of intermetallic coatings for analysis by scanning transmission electron microscopy (STEM), atomic force microscopy (AFM), and ToF-SIMS. Excellent isotopic detection of light elements was illustrated using ToF-SIMS. For example, ToF-SIMS’s excellent mass resolution and sensitivity allowed for the analysis of light elemental isotopes such as hydrogen/deuterium and ^6^Li/^7^Li. Figure 7 presents a multimodal analysis workflow and attainment of information from each technique, which was selected due to the complementary information over an area when compared to bulk techniques on irradiated samples.

**Figure 8:**
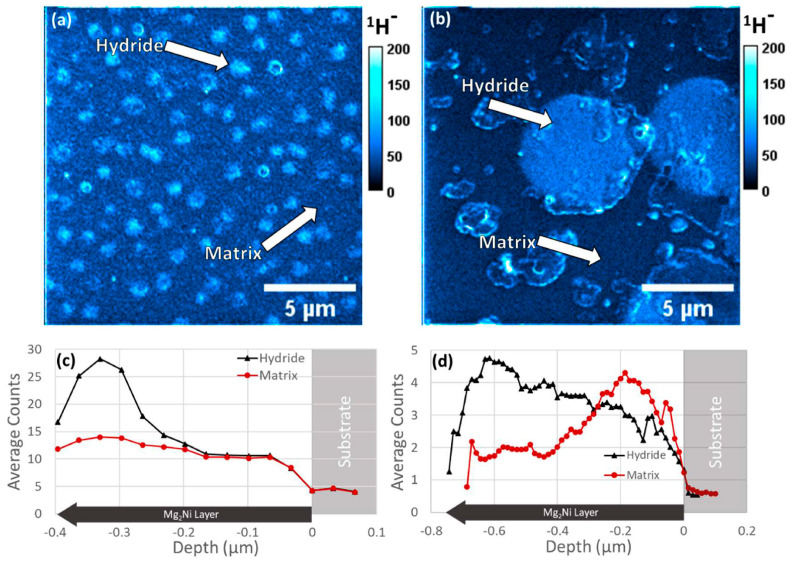
SIMS images with (**a**) equiaxed microstructure showing the summed signal over three slices and (**b**) columnar microstructure showing the summed signal over 14 slices. (**c**,**d**) SIMS localized depth profiles from regions inside and outside of the surface visible hydride areas for (**a**) and (**b**), respectively, were reproduced with permission from Ref. [101]. Copyright 2023 Elsevier.

SIMS allowed for a complex investigation of possible lithium mobility within the sample via both spectral analysis and depth profiling. SIMS spectral analysis identified hydrogen, deuterium, and tritium presence, as well as ^6^Li and ^7^Li. It was also observed that tritium is deposited on the cladding coating and has a large possibility of being a product during irradiation due to tritium levels being far larger than the natural abundance. Depth profile measurements for Li suggested that it is associated with alumina oxide layers, and tritium signals were much less intense in the middling depths of the cladding. Andersen et al. studied hydrogen inclusion using magnetic sector SIMS [101]. Mg_2_Ni/Mg_2_NiH_4_ thin films were analyzed with high-resolution imaging and depth profiling to characterize such materials in fields from batteries to high-strength alloys using magnetic sector SIMS [131,152]. Both images and depth profiles of a Mg_2_Ni film can be found in Figure 8. In this regard, SIMS imaging bridged the gap between TEM and X-ray diffraction (XRD), allowing for the 3D chemical measurement of hydrogen with a resolution of tens of nanometers and a field of view (FOV) of tens of microns.

SIMS and EBSD were combined to study polycrystalline nickel and to investigate hydrogen distribution around grain boundaries to see the effect on the grain boundaries [153]. EBSD inverse pole figure mapping was combined with hydrogen concentration profile mapping from SIMS. This multimodal imaging strategy showed two different types of hydrogen distribution behavior in nickel. The first is categorized by fast hydrogen diffusivity and showed a sharp gap for hydrogen concentration profiles across random grain boundaries. The second category is across special grain boundaries, characterized by low hydrogen diffusivity.

#### 4.5.2. Semiconductors Using

Semiconductors are a type of material structure that can benefit from multimodal SIMS strategies, as small optimizations in composition can lead to drastic changes in observed properties. Usiobo et al. applied Helium-ion Microscopy (HIM) coupled with SIMS to study mixed organic and mixed halide perovskite semiconductors [124]. These perovskite semiconductors are used for solar cell devices, and continued efforts have been made to reduce the instability in certain environments through doping. Alkali cation pairs such as K-Cs, K-Rb, and Rb-Cs were analyzed, allowing for both elemental and morphological imaging at the nanometer scale. Correlative imaging permits the characterization of chemical content, distribution of grains, and secondary phases. The fusion of imaging techniques allowed for combinatory structural images and chemical maps. Results showed that Rb accumulates at the semiconductor’s grain boundaries while still having a presence within perovskite grains regardless of the cation pairing chosen.

Kumar et al. employed SEM, TEM, and NanoSIMS to study Si-metal interfaces of screen-printed solar cells, which is a primary source for the cell’s loss of efficiency. NanoSIMS was selected for inclusion as dopant levels within the cells were reported to be below the 0.1 wt.% detection limit of conventional analytical techniques such as EDX. NanoSIMS-enabled dopant distributions were imaged, and SEM allowed for analysis of phases present within the sample. It was discovered that phosphorus-emitting structures, identified by NanoSIMS, and SiN_x_ passivation layers were destroyed if the cells were overfired, which was validated in the correlated SEM analysis. These results point towards diffusion of the dopant species, lowering the overall cell efficiency due to these microstructural losses.

The doping of SI nanocrystals has long been hindered by the separation of theoretical calculations, where thermodynamic equilibrium conditions are usually utilized, and experimental conditions, where nanocrystal incorporation is common. Perego et al. used a multimodal technique to study P-doped Si nanocrystals embedded in SiO_2_ to allow for the understanding of kinetics while not being directly tied to equilibrium conditions [154]. Energy-filtered transmission electron microscopy (EF-TEM) cross-sectional images were obtained, along with ToF-SIMS depth profiling, Rutherford Backscattering Spectrometry (RBS), X-ray photoelectron spectroscopy (XPS), and nuclear reaction analysis (NRA). XPS allowed observation of the diffused P trapped in the nanocrystals and incorporated either in the nanostructure’s core or in an interface region. XPS’s 1000–2000 ppm was insufficient to detect P levels in the surrounding SiO_2_. ToF-SIMS depth profiling was coupled with TEM cross-sectional images to compare nanocrystal size distribution before and after annealing. The data were compared to diffusion models to confirm diffusion behavior. It was estimated that the P content in the matrix was a fraction of that contained within the embedded nanocrystals. SIMS analysis at various annealing temperatures provided information into the dynamics of the trapping behavior. Further results revealed that high P concentration in Si nanocrystals embedded in SiO_2_ corresponded to a thermodynamically favored system configuration, with six times the solubility in the bulk material. P trapping behavior in the embedded nanocrystals was shown to be limited by diffusion, lacking additional diffusion barriers. It was, therefore, proven possible for high levels of impurities to be introduced into the inner layers of Si nanocrystals with dopant properties finely tunable with changing annealing conditions. This approach could be particularly appealing in conjunction with monolayer doping processes to control dopants introduced in nanostructured systems.

#### 4.5.3. SIMS Complementary Techniques

SIMS imaging can be applied alongside a wide range of techniques, providing complementary information for a more well-rounded analysis. Otto et al. employed XPS and ToF-SIMS to better understand the passivation layer of Li-metal interfaces [155]. Employing XPS for its quantitative element and compound-specific information, SIMS was able to boost the low lateral and depth resolutions of XPS as well as increase the sensitivity of Li and the detection of H. Results showed that the Li passivation layer was mainly homogenous with contaminant presence. A bi-layered structure of a hydroxide and carbonate layer was reported atop an oxide-rich region. The multi-analytical approach was required for a comprehensive characterization of the film. XPS’s quantitative compositional data provided the sequencing information, and ToF-SIMS allowed for depth measurements of the layer thickness, distribution, and homogeneity. Amadelli et al. similarly paired XPS and SIMS to study PbO_2_ on Ti when electrodeposited [156]. Results showed from the complementary techniques that the dopant species affect the accumulating behavior of O species at the oxide surfaces. F^−^ was found to be incorporated into the PbO_2_, and the presence of cations, such as Fe_3_^+^, Ni_2_^+^, and Co_2_^+^, was not found in the coatings, even when added to the used solutions. Kellner et al. employed a multivariate analytical approach to analyze V and Cr-containing metal alloys [157]. An EDX system measured chemical composition, and TEM/STEM measurements allowed for the distributions of elements and compounds when coupled with EDX results. SIMS was able to support and correlate these results by presenting distribution information of its own, providing insight into the presence of V and Cr. Along with TEM, the results described the effects of V and Cr on the corrosion process. Results showed Cr additions gave better corrosion resistance than V, providing a passivation layer. Grovenor et al. applied SIMS, APT, and TEM to study the oxidation mechanisms of Zr fuel cladding alloys [158]. Previous EDX results had shown the oxide layers of Zr fuel cladding to include an intermediate oxide layer. APT revealed these suboxide layers within the cladding, and TEM validated these measurements. SIMS was employed to demonstrate the penetrating ability of selected oxidizing species through outer oxide layers. SIMS measurements tracked the specific portions of oxide that were active in the oxidation process during corrosion. In addition, results demonstrated that the transition between the corrosion environment was located at the metal/oxide interface, using the porosity of the layers observed with other techniques as routes of access.

### 4.6. Nanomaterials 

A specific subset of SIMS applications is in the rising field of nanomaterials. The scope of nanoparticles and nanostructured alloys has been increasing over recent decades due to the opportunity to obtain properties not available in contemporary materials’ equivalents. These materials require reliable and effective tools for characterization to optimize novel systems and processes effectively. SIMS sensitivity and excellent resolution allow for it to serve as a linchpin in the effective analysis of nanomaterials. 

Priebe et al. used a collection of TEM and SIMS analysis techniques to study Al nanoparticles in a ZrCuAg matrix [142]. SIMS was selected for analysis to study elemental composition, while TEM provided nanoparticle size measurements. The objective of these complementary techniques was to characterize nanoparticles and to aid in optimizing the nanocomposite’s properties for medicinal applications. The result was a successful attempt at spatially resolving nanoparticles in an inorganic matrix, using SIMS to provide elemental information and TEM to validate SIMS in determining nanoparticle sizes. Tian et al. use SIMS to analyze Mg–Gd–Y–Zr alloys with continuous gradients and nanograin sizes to observe if solute clustering contributed to alloy strengthening [159]. SIMS imaging and depth profiling were employed with SEM and EDX. Results showed an even distribution of alloying elements, with little clustering at the surface, confirming that a solid solution contributes most to strengthening behaviors rather than precipitation. Interface/surface segregation was also observed by SIMS; however, it did not exceed the component’s maximum solubility. Choi et al. analyzed a nanostructured Ni-based alloy conjoined via dissimilar weld joints with low alloy steel using SIMS, APT, and TEM [160]. SIMS was employed to construct the chemical map of the alloy constituents at the weld sites, TEM was used to analyze the transition of crystallographic microstructure, and APT was used to determine the chemical composition of specific boundary regions. Their findings suggest the nano-precipitate distribution to be uneven across the bulk, and the weld region to be divided into several areas including an unmixed zone in the Ni-based nano-alloy, a fusion boundary, and a heat affected zone in the steel. This non-homogeneous distribution included interesting observations, such as higher Fe and lower Mn, Ni, and Cr from the low alloy steel compared to the filler metal utilized, with carbide precipitation near the weld fusion boundary.

## 5. SIMS Data Challenge

### 5.1. Multivariate Analysis (MVA)

A major challenge associated with the application of SIMS is the complexity of datasets, especially for ToF-SIMS. It has big data challenges due to the nature of parallel data collection [4,161,162]. For ToF-SIMS analysis, multivariate analysis (MVA) is often used. MVA encompasses a set of mathematical techniques designed to simplify and dissect these complex SIMS datasets. Principal Component Analysis (PCA) is the most frequently employed method among MVA methods, as it effectively reduces the dimensionality of large SIMS datasets, thereby highlighting the most significant variates or characteristic peaks. MVA offers three distinct advantages when compared to traditional analysis methods. First and foremost, it presents an objective and statistically reliable approach, minimizing potential bias by eliminating the need for manual selection of specific peaks for analysis. Second, it enhances the signal-to-noise ratio by considering all available information in the dataset. Third, it is often fast and automated, with a typical analysis taking only a few minutes on a modern desktop computer. PCA has already demonstrated successful applications in characterizing and quantifying a wide range of materials, including inorganic materials [163,164,165], polymers [166,167], polymer additives [168], organic thin films [169,170,171], proteins [172,173,174], self-assembled monolayers [175], and bacteria samples [176,177].

Typically, when conducting PCA, the analyzed dataset is pre-segmented into several a priori-defined subgroups, often stemming from the experimental study design, such as control groups and treatment-specific sample groups. The data are usually subjected to normalization based on the total ion intensities of selected peaks, square-root transformation, and mean centering before PCA is executed [6,53,177,178,179,180,181]. When evaluating the PCA results, scores plots and loadings plots are commonly presented together. Score plots illustrate the similarity and dissimilarity among samples while loading plots reveal the contributions of components corresponding to scores in the principal component (PC). Peaks with high loadings contribute more significantly to the observed clustering of peaks in the scores plot [53,180]. The first principal component (PC1) represents the maximum possible variation in the dataset, with the second principal component (PC2) accounting for the maximum variation in uncorrelated data with PC1. In this interactive manner, all variations in the dataset are captured by the derived PCs. Typically, the first few PCs encapsulate the majority of the variation in the dataset when the original variables exhibit inter-correlation. For more in-depth information, interested readers are encouraged to refer to the original work by Jolliffe (1986) for a detailed description of this technique [182].

Multivariate curve resolution (MCR) represents another frequently employed approach that, much like PCA, aims to maximize the explained variance in complex datasets. This technique has found widespread use in the interpretation of mass spectrometry datasets [183,184]. When compared to PCA, MCR offers several advantages [185]. For instance, MCR factors need not be mutually orthogonal. By applying non-negativity constraints to the loadings and scores matrices during the optimization process, MCR solutions closely resemble ToF-SIMS spectra and chemical contributions, as these inherently possess positive values. However, it is important to note that MCR is computationally more intensive than PCA, and it demands more extensive input prior to analysis. Additionally, MCR generates solutions that are not unique; they are reliant on initial estimates, constraints, and convergence criteria. Consequently, a cautious approach is necessary to yield optimal results. For additional insights, readers can refer to Gallagher et al.’s introduction to MCR with applications to ToF-SIMS [186]. A comprehensive overview of the technique can also be found in the reference provided by de Juan et al. in 2003 [187]. Other multivariate analysis methods, such as maximum autocorrelation factors (MAF), discriminant analysis (DA), partial least squares (PLS), and cluster analysis, have also been employed to reveal information in complex ToF-SIMS datasets [188].

### 5.2. ML to Address SIMS Data Challenge

The difficulty in SIMS analysis due to the complex factors that contribute to the collected data, as well as the quantity of data collected, makes SIMS desirable for the application of machine learning (ML). ML strategies help to alleviate the strain caused by large volumes of data [189]. MVA takes advantage of unsupervised methods, such as PCA and non-negative matrix factorization (NMF) [190,191]. Logistic regression, a classical model that determines the probability that a given model belongs to a certain class, is also among the common methods employed, with low cost and easy implementation [192].

Heller et al. illustrate an excellent example of how AI and ML can aid in the challenge of SIMS data analysis [193]. MVA was applied to an unknown surface composition of an aged lithium battery anode due to its large amounts of unidentified degradation products, which complicated manual processing [193]. PCA was applied to find relevant peaks within the ToF-SIMS spectra, with >75% of characteristic peaks that were previously unknown identified. MCR was then applied to the depth profiles of the samples, and the layered structure was discovered. PCA was shown to be applicable to studying all layer compositions simultaneously. The MVA analysis allowed for the study of different compounds from the mass spectra to aid in determining the degradation products in the aged lithium. The usage of dimensional reduction techniques with physical and chemical constraints presents another useful strategy for reducing analytical complexity for SIMS techniques [194].

Lombardo et al. applied ML to Li-ion battery electrode microstructures by mapping both main phases and degradation products [195]. This method identified and characterized single particles through a watershed-based slicing algorithm, which segments objects in images apart [196]. The segmented and identified particle images trained an ML algorithm that reconstructs 3D microstructures from 2D images.

Since Li-ion electrodes’ properties are dependent not only on the fractions of materials used but also on the distribution of those elements’ interfaces, ToF-SIMS sensitivity, and FOV allow for an excellent basis of study, and ML is an excellent collaborative technique to streamline the process of 3D imaging of interfacial boundaries. Segmented images were used to train an ML algorithm that permitted 3D microstructure reconstruction from 2D inputs. This process allowed for the mapping of interphase locations instead of the distribution of the contained species, including the possibility of mapping the degradation products. LiCO_3_ was among the best choices for mapping cathode electrolyte interphases. The ML-enhanced 3D mapping reveals that P-rich cathode electrolyte interphases were found in regions subjected to high electronic current densities and carbonate-rich cathode electrolyte interphases in regions with higher ionic current densities.

Griffin et al. employed the dimensional reduction ML technique to assess the nanoscale relaxation response of a solid solution, allowing for a comparison of composition-dependent behaviors [194]. A poling and relaxation behavior was identified, and the evolution of those behaviors was tracked alongside the phase diagram of the materials. This reductive strategy was applied to ToF-SIMS by Abbassi et al., with four LaAlO_3_ and SrAlO_3_ heterostructures studied through PCA and NMF [197]. It was shown that the strategy provided dimensional stacking statistics while maintaining the separability of the different specimens. The four samples were studied with NMF and PCA before and after stacking to compare the advantages of the technique. NMF showed that separation of all sections of the film (surface, film, interface, and substrate) was possible. Even with stacking, the analysis was still able to detect the composition of the film as AlO and AlO_2_. The surface analysis of those films, where no sputtering occurred, showed cations including La. Dimensional stacking, and reduction, allowed for the identification of the Al, O associated surface layer of the four structures.

**Figure 9:**
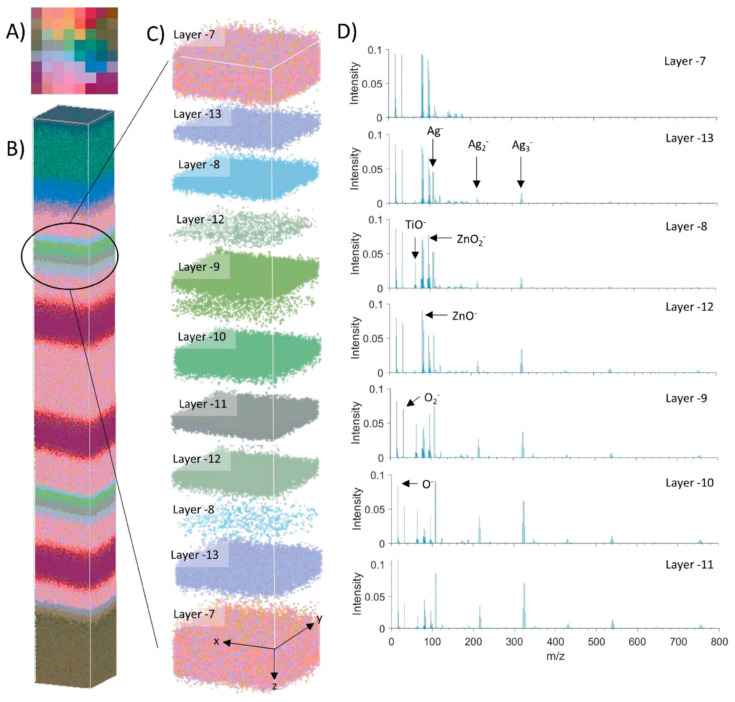
Negative ion ToF-SIMS depth profile (Cs^+^ sputter beam) of low-E double silver glass coating. (**A**) SOM indicating colored clusters, containing 64 neurons. (**B**) Three-dimensional visualization of depth profile indicating similarity using SOM-RPM model of the negative polarity data. (**C**) Reconstruction of the 3D layer structure of the upper silver region. (**D**) Average ToF-SIMS spectra from each layer in (**C**). Different colors are used to indicate layers. Reproduced with permission from Ref. [83]. Copyright 2023 Wiley Online Library.

Multilayer coatings are a common application space for ML to aid in data analytics, as compositions changing with the layer depth increases the complexity of characterization. Bramford et al. illustrated the use of ML and ToF-SIMS in the 3D chemical characterization of silver coating on glass [83]. These coatings were stated to help increase the energy efficiency of buildings using complex multilayer film stacks with each layer employed in a particular function (i.e., anti-reflective coatings, mechanical protection). Positive and negative ToF-SIMS data were collected, and self-organizing maps with relational perspective mapping (SOM-RPM) were applied to the data. This ML approach models chemical similarities by tagging each pixel with a color. The original position of each pixel is then returned, and a similarity map is generated where the color represents the similarity to other pixels. This strategy allows for the visualization of large volumes of data and 3D regions and can be seen in Figure 9. This technique was used to show the chemical similarity between differing layers of a silver film. Repeating layers were identified and classified as chemically indistinguishable based on the entire gathered ToF-SIMS mass spectra. SnO_2_ dielectrics, ZnO seeding layers, TiO_x_ blocking layers, a Zn base layer, and a TiO_x_ topcoat were all identified. Chemical changes were detected with depth in the optical silver layer. This ML technique was shown to provide insight into both manufacturing processes and production challenges due to the simplification of data analysis for otherwise complex materials.

## 6. Outlook and Recommendations

This review serves to highlight the role that SIMS has played in the analysis of metals and alloys. A summary of the history and recent developments of different SIMS techniques is given. SIMS principles, differing analyzers, and instrument parameters of various instruments are summarized. SIMS’s measurement modalities are described along with the limitations of the techniques. Applications of several major SIMS types are given by fields and subjects that commonly utilize a particular technique. Applications of SIMS inclusion in correlative imaging techniques seek to show SIMS’s capacity for collaborative analysis. The volume and complexity of SIMS data are explored, and efforts utilizing ML are emphasized as ongoing efforts to manage the big data challenge that SIMS brings.

SIMS surface sensitivity, detection limits, and ability to analyze isotopes and all elements provide a very diverse range of applications across many differing fields. SIMS spectral analysis and depth profiling allow for the distribution of all elements at low concentrations to be spatially resolved, providing information on microstructural features such as layer interfaces and segregated/enriched phases. SIMS’s excellent resolution and sensitivity, coupled with its ability to provide both spectral and depth-resolved information, make it a powerful tool in the characterization and analysis of metals and alloys.

Further studies may be greatly improved using in-situ and operando SIMS. The capacity to provide real-time analysis of surfaces under active transformation allows for a better understanding of short-lived intermediate-stage behaviors/phases [6,52,56]. This would greatly aid in fields where understanding dynamic processes is paramount, such as corrosion evolutions and catalyst behavior. The utilization of SIMS in multimodal analysis environments is a promising field where other macro- and micro-analysis techniques can be integrated to obtain unique insights into the various materials of study while compensating for each technique’s limitations. These multimodal studies directly demonstrate the advantages that SIMS has and the role it can play in multifaceted analysis. The SIMS approach complements many material microanalysis techniques, including XPS, TEM, or SEM, to name a small selection. SIMS’s versatility in obtaining surface information can provide opportunities to present more well-rounded analysis conclusions than using only select techniques on their own. Depth profiling and spectral analysis are excellent tools for the of light isotopes such as deuterium and have the possibility to allow SIMS to take a more central role in investigating these microstructural elements. Many fields find SIMS’s challenges in data analysis difficult to overcome. The possibilities for AI and ML to be applied to SIMS is an area with many possibilities for future work. The potential to reduce the dimensionality of large datasets allow for the alleviation of complex material compositions or large quantities of data. Further improvements to such algorithms and the creation of new ML models and pathways would allow for high throughput SIMS analysis.

## Figures and Tables

**Table 1:** Comparison of mass analyzers used in SIMS.

Mass Analyzer	Resolution	Practical Mass Range	Transmission	Mass Detection	Relative Sensitivity
Quadrupole	10^2^–10^3^	<10^3^	0.01–0.1	Sequential	1
Magnetic sector	10^4^	>10^4^	0.1–0.5	Sequential	10
Time-of-flight	100,000	10^3^–10^4^	>0.5	Parallel	10^4^

## Data Availability

No new data were created or analyzed in this study. Data sharing is not applicable to this article.

## Glossary

Al	Aluminum
APT	Atom Probe Tomography
B	Boron
CIGS	Cu(In,Ga)Se_2_
Cu	Copper
EDX	Energy-Dispersive X-ray
EF-TEM	Energy Filtered Transmission Electron Microscopy
FIB	Focused Ion Beam
FOV	Field of View
FTIR	Fourier Transform Infrared
HIM	Helium Ion Microscopy
Li	Lithium
ML	Machine Learning
MVA	Multivariate Analysis
MCR	Multivariate Curve Resolution
NMF	Non-Negative Matrix Factorization
NRA	Nuclear Reaction Analysis
PEELS	Parallel Electron Energy Loss Spectroscopy
PCA	Principal Component Analysis
P	Phosphorus
RBS	Rutherford Backscattering Spectrometry
SECM	Scanning Electrochemical microscopy
SEM	Scanning Electron Microscopy
STEM	Scanning Transmission Electron Microscopy
SIMS	Secondary Ion Mass Spectrometry
SHRIMP	Sensitive High-Resolution Ion Microprobe
ToF	Time-of-Flight
TEM	Transmission Electron Microscopy
V	Vanadium
XRD	X-ray Diffraction
XPS	X-ray Photoelectron Spectroscopy
